# Effects of adiposity on the human plasma proteome: Observational and Mendelian randomization estimates

**DOI:** 10.1038/s41366-021-00896-1

**Published:** 2021-07-05

**Authors:** Lucy J. Goudswaard, Joshua A. Bell, David A. Hughes, Laura J. Corbin, Klaudia Walter, George Davey Smith, Nicole Soranzo, John Danesh, Emanuele Di Angelantonio, Willem H. Ouwehand, Nicholas A. Watkins, David J. Roberts, Adam S. Butterworth, Ingeborg Hers, Nicholas J. Timpson

**Affiliations:** 1 Medical Research Council (MRC) Integrative Epidemiology Unit at the University of Bristol’ Bristol’ UK; 2 Population Health Sciences, Bristol Medical School, University of Bristol, Bristol, UK; 3 School of Physiology, Pharmacology and Neuroscience, University of Bristol, UK; 4 Bristol Heart Institute, Bristol, UK; 5 Wellcome Sanger Institute, Hinxton UK; 6 Department of Haematology, School of Clinical Medicine, University of Cambridge, UK; 7 MRC/BHF Cardiovascular Epidemiology Unit, Department of Public Health and Primary Care, University of Cambridge, Cambridge, United Kingdom; 8 NIHR Blood and Transplant Research Unit in Donor Health and Genomics, Department of Public Health and Primary Care, University of Cambridge, Cambridge, UK; 9 British Heart Foundation Centre of Research Excellence, University of Cambridge, Cambridge, UK; 10 NIHR Cambridge Biomedical Research Centre, University of Cambridge and Cambridge University Hospitals, Cambridge, UK; 11 Health Data Research UK Cambridge, Wellcome Genome Campus and University of Cambridge, Cambridge, UK; 12 NHS Blood and Transplant, Cambridge Biomedical Campus, Long Road, Cambridge, UK; 13 NHS Blood and Transplant-Oxford Centre, Level 2, John Radcliffe Hospital, Oxford, UK; 14 Radcliffe Department of Medicine, University of Oxford, John Radcliffe Hospital, Oxford, UK

## Abstract

**Background:**

Variation in adiposity is associated with cardiometabolic disease outcomes, but mechanisms leading from this exposure to disease are unclear. This study aimed to estimate effects of body mass index (BMI) on an extensive set of circulating proteins.

**Methods:**

We used SomaLogic proteomic data from up to 2 737 healthy participants from the INTERVAL study. Associations between self-reported BMI and 3 622 unique plasma proteins were explored using linear regression. These were complemented by Mendelian randomization (MR) analyses using a genetic risk score (GRS) comprised of 654 BMI-associated polymorphisms from a recent genome-wide association study (GWAS) of adult BMI. A disease enrichment analysis was performed using DAVID Bioinformatics 6.8 for proteins which were altered by BMI.

**Results:**

Observationally, BMI was associated with 1 576 proteins (P<1.4x10^-5^), with particularly strong evidence for a positive association with leptin and fatty acid-binding protein-4 (FABP4), and a negative association with sex hormone binding globulin (SHBG). Observational estimates were likely confounded, but the GRS for BMI did not associate with measured confounders. MR analyses provided evidence for a causal relationship between BMI and eight proteins including leptin (0.63 standard deviation (SD) per SD BMI, 95% CI 0.48-0.79, P=1.6x10^-15^), FABP4 (0.64 SD per SD BMI, 95% CI 0.46-0.83, P=6.7x10^-12^) and SHBG (-0.45 SD per SD BMI, 95% CI -0.65 to -0.25, P = 1.4x10^-5^). There was agreement in the magnitude of observational and MR estimates (R^2^ = 0.33) and evidence that proteins most strongly altered by BMI were enriched for genes involved in cardiovascular disease.

**Conclusions:**

This study provides evidence for a broad impact of adiposity on the human proteome. Proteins strongly altered by BMI include those involved in regulating appetite, sex hormones, and inflammation; such proteins are also enriched for cardiovascular disease-related genes. Altogether, results help focus attention onto new proteomic signatures of obesity-related disease.

## Introduction

Obesity has tripled worldwide since 1975, now affecting around 40% of adults in the United States and 26% of adults in the UK (1). The average body mass index (BMI) of the UK adult population is now in the conventional ‘overweight’ category (BMI between 25 and 30 kg/m^2^) (2) and ‘overweight’ is now more common than ‘normal-weight, in middle age in many high-income countries (3). BMI is often used as a proxy for adiposity given high correlations between BMI and more objectively measured fat mass indices (4). Higher adiposity is a major risk factor for various noncommunicable diseases including type II diabetes, cardiovascular diseases, musculoskeletal diseases, and cancer (5–8), which collectively put a strain on health services (9, 10). These BMI-disease associations are supported by prospective observational studies and, more recently, by Mendelian randomization (MR) studies (11–13), which use genetic variation reliably associated with BMI to re-estimate effects of BMI on disease outcomes. Given the properties of genetic variation, this method helps to overcome issues such as confounding and reverse causation which commonly occur with observational studies (14).

Despite MR studies supporting a causal role of adiposity for cardiometabolic diseases, and randomised trials supporting the effectiveness of weight loss in reducing disease risk (15), the molecular footprint of adiposity is not well understood. Previous studies have largely focused on the impact of higher BMI on the lipidome including traits such as cholesterol and triglycerides in lipoprotein subtypes (e.g. low-density lipoprotein (LDL) and high-density lipoprotein (HDL) particles) (4, 16), and on inflammatory molecules such as C-reactive protein (CRP) (16, 17).

A benefit of studying the systematic effects of BMI on the circulating proteome is that proteins are often more suitable pharmacological targets than metabolites. Efforts to study the effect of BMI on the proteome have generally been in an observational framework (18). It is estimated that 25% of proteins in the human proteome circulate in blood (19), which is important as the majority of druggable targets are such proteins (20). Studying the effect of BMI on a large set of proteins has only recently become possible with newly developed proteomic technologies such as the SomaLogic platform, with the ability to quantify enzymes, protein kinases and transport proteins with unprecedented sensitivity (21). Utilisation of SomaLogic within a trial or cohort setting has recently become more widespread, such as within the INTERVAL study, a UK cohort of blood donors (22). There is evidence that proteins which change as a result of a higher BMI may contribute to cardiometabolic disease (23): identification of such proteins is important in understanding how higher BMI causes disease and to identify targets which may benefit from pharmacological intervention.

In this study, we aimed to measure associations between adiposity and the human proteome and to also estimate the underlying effects in a causal framework. Using data on 2 737 participants from INTERVAL, we estimated effects of BMI on 4 034 (3 622 unique) plasma protein traits, in both observational and MR frameworks. We examined the agreement between effect estimates from different methods and performed enrichment analyses of the most strongly altered proteins to map their potential relevance to disease.

## Methods

### Study population

INTERVAL is a prospective cohort study which was initially a randomised trial that aimed to test the efficiency and safety of reducing the time between whole blood donation in approximately 50 000 participants (24). Upon informed consent, eligible participants who were: aged 18 years and over, willing to complete online questionnaires and without a self-reported history of major disease were recruited between June 11^th^, 2012 and June 15^th^, 2014 from 25 National Health Service Blood and Transplant (NHSBT) centres across England. Participants filled out questionnaires including self-reported height and weight, smoking frequency and alcohol consumption. Blood samples were taken at baseline which were analysed for full blood counts and blood biomarkers. This study was approved by Cambridge (East) Research Ethics Committee. Access to the data was granted by the Data Access Committee.

The present study was conducted on a random subset of participants from INTERVAL who had basic phenotype data and plasma proteins measured by SomaLogic. This included up to 2 737 participants mostly of European descent across analyses described below.

### Assessment of BMI and covariables

Participants completed online questionnaires wherein they reported their height and weight. BMI was calculated as weight in kilograms divided by the square of their height in metres (kg/m^2^). Available covariables were age, sex, previous or current smoking frequency (in three categories of: never, occasional, most days or every day) and alcohol intake frequency (in four categories of: rarely, less than once a week, 1-2 times a week, 3-5 times a week or most days). These covariables were chosen as they were measured in the INTERVAL collection and are measures which are thought to influence adiposity and cardiometabolic health (4).

### Measurement of circulating proteins

Plasma proteins were measured in INTERVAL participants at baseline (before randomisation of assignment to the time interval between blood donation) using the SomaScan® by SomaLogic (22). This platform uses 4 034 modified nucleotides known as Slow Off-rate Modified Aptamers (SOMAmers) which make direct contact with proteins, enabling detection of 3 622 unique proteins or protein complexes and quantifies them using a DNA microarray (21). Separate SOMAmers can bind to isoforms of the same protein, but can also bind to the same protein at different sites (which can be impacted by post-translational modifications or complexes formed with other proteins). We therefore have included all 4 034 SOMAmers. The extensive number of proteins measured, with no missingness and in a cohort of 2 737 participants, provides a rich proteomic dataset. The proteins were measured in relative fluorescence units (RFUs) and quality control (QC) was performed as described by Sun et al. (22). There was no missingness across protein variables. The proteomic data used had been pre-adjusted (using linear regression) for age, sex, duration between blood draw and sample processing (1 day or less vs >1 day), and the first three genetic principal components, with the residuals inverse normal rank transformed. All following analyses use this “pre-adjusted” data as input.

### Genetic data and instrument for BMI

INTERVAL participant genotyping was performed on the Affymetrix GeneTitan® Multi-Channel (MC) Instrument using the UK Biobank Axiom^™^ Array (ThermoFisher Scientific, Loughborough, UK) and the QC of genotype data was implemented as described by Astle et al. (25). The imputation panel used was the 1 000 genomes phase-3-UK-10K (25). A genetic instrument for BMI was constructed using 654 genetic variants that were associated with BMI at P<5x10^-8^ in the inverse variance weighted fixed-effect meta-analysis of GWAS of ~700 000 individuals of European ancestry (26). This meta-analysis consisted of ~250 000 adults from the Genetic Investigation of ANthromopetric Traits (GIANT) consortium (27) and ~450 000 adults from the UK Biobank study. Only 0.05% of UK Biobank participants were included in the current INTERVAL study (of N=2 737). These participants were not excluded to increase power. The weighted GRS was made using PLINK 2.0 software (28) using the effect alleles and beta coefficients from the source GWAS. The score was calculated by multiplying the number of effect alleles at each SNP by its effect estimate (beta), summing these, and dividing by the total number of SNPs included. The GRS therefore can be interpreted as the average per-SNP effect on BMI for each individual.

### Statistical analyses

The population characteristics of INTERVAL participants with SomaLogic data who were included in this study (N range: 2 422 to 2 737 due to missing data for covariables) were compared to those INTERVAL participants who were not included (N range: 27 174 to 30 721) to assess generalisability of any BMI-protein associations to the wider INTERVAL sample. Population characteristics evaluated were age, sex, weight, height, BMI, smoking frequency, and alcohol intake. Differences in population characteristics among the two INTERVAL sub-sets were tested by a two-sided t-test for continuous traits and a two-sided Chi-square test for categorical variables. Observational analyses were conducted using linear regression to examine associations between BMI (in normalized standard deviation (SD) units based on a rank normal transformation (*rntransform()* from “moosefun” package https://github.com/hughesevoanth/moosefun) and each standardised protein trait as a dependent (outcome) variable. Two linear models were used (using *lm()* function from R “stats” package): 1) adjusted for age and sex and 2) additionally adjusted for smoking and alcohol consumption (each as an ordered categorical variable). Given that the procedure which generates the “pre-adjusted” data (adjustment for covariables before rank normal transformation of proteins) can reintroduce correlations (29), age and sex are again used as covariables here. The estimates derived from models 1) and 2) therefore reflect the normalized SD-unit difference in each protein trait per normalized SD-unit (4.8 kg/m^2^) higher BMI. Associations of covariables with BMI and protein traits were also examined using linear regression.

A Shapiro-Wilk test was used to confirm whether the GRS showed a normal distribution. MR analyses were conducted using two-stage least squares (2SLS) regression models with robust standard errors, using the systemfit function as part of the systemfit package (30), with measured BMI in SD units and the GRS for BMI as the instrumental variable. These MR estimates reflect the normalized SD-unit difference in each protein trait per normalized SD-unit (4.8 kg/m^2^) higher BMI. We report estimates from the direct linear associations between BMI and proteins as “observational” estimates and those from the 2SLS causal effect estimates as “MR estimates”. Agreement between observational and MR estimates was examined using a separate linear regression. This was performed: 1) for all proteins, and 2) excluding the proteins that fell below our P-value reference point for strong evidence (defined below) to examine whether agreement is limited to ‘top hits’ or applies throughout the effect distribution. Agreement between observational estimates and MR estimates would suggest that there are causal effects of BMI across the general proteome, with differences in estimates suggesting confounding of observational estimates.

To account for multiple testing, a Bonferroni correction was used to adjust results. This was informed by the correlation between proteins, adjusting only for the estimated number of independent traits (Supplementary Figure 1). Correlation was assessed by a Spearman’s correlation matrix. From a starting number of 4 034, the number of independent proteins was 3 655 (using a correlation cut-off of r = 0.8 or tree cut height = 0.2 between proteins, Supplementary Figure 2, dendrogram made using “iPVs” package https://github.com/hughesevoanth/iPVs). We utilised a Bonferroni adjusted P-value of 0.05/3655 = 1.4x10^-5^ to indicate strong evidence in this sample. Full results are presented in the supplementary material.

### Enrichment analysis

To investigate whether any protein groups showed a particularly strong relationship with BMI and disease (signal detection), protein features were clustered for further analysis. First, a principal component analysis (PCA; *prcomp*() function from the R “stats” package) on the proteins, not the individuals, was performed on the “pre-adjusted” (see above) dataset (Supplementary Figure 3A-D). The top ‘n’ PC eigenvectors, as identified by a scree plot of the PCA eigenvalues (Supplementary Figure 3E), were carried forward into an unsupervised k-means analysis *(kmeans()* function from the R “stats” package). Nineteen k-means analyses were run altering the value of k (number of clusters) from two to 20. To identify an appropriate number of protein clusters (k) we generated a scree like plot (Supplementary Figure 3F). Here we plotted the variance explained by clusters, for each k, as estimated as the sum of squares explained by clusters (betweenness) over the total sums of squares, and looked for the smallest k with the maximum variance explained (a plateau). In summary, we used a data reduction method (PCA) to identify major axes (PCs) of the protein data that were then utilised in a machine learning clustering algorithm (k-means) to identify clusters of proteins that share abundance similarities across individuals.

To explore whether there was a systematic difference in the association of proteins within these clusters and BMI, the beta coefficients from the observational linear regressions or MR models were transformed into their absolute values and divided by their standard error (SE). The absolute betas divided by their SEs in each cluster was compared using a one-tailed pairwise Wilcox test to identify which clusters showed a stronger association with BMI. For the cluster(s) showing evidence for larger absolute beta coefficients, an enrichment analysis was performed using DAVID bioinformatics resources 6.8 (31). Enrichment was assessed by using the uniprot IDs for the proteins in the cluster and comparing these proteins with the uniprot IDs of the full SomaLogic protein list. Enrichment for protein involvement in disease (using the genetic association database (GAD) disease classes (32)) of the protein cluster was assessed by fold enrichment and a Bonferroni-corrected P-value to account for multiple testing. Proteins that were associated with BMI in confounder-adjusted observational analyses at P<1.4x10^-5^ were also entered into the disease enrichment tool and compared with the total proteins (as described for cluster enrichment). Analyses were performed using R version 3.4.2 (33). R code used for analyses available upon request.

## Results

### Participant characteristics

INTERVAL participants included in this study (those with proteomic data), had a mean age of 45.0 years (SD of 14.1 years) and 48.3% were female (Table 1). Mean BMI was 25.9 kg/m^2^ (SD of 4.8 kg/m^2^) and the majority of participants were non-smokers (59.1%). Nearly a quarter (23.5%) reported currently or previously smoking daily and 71.5% reported drinking alcohol at least once a week. Participants with proteomic data were representative of the full INTERVAL cohort (Supplementary Table 1).

### Observational estimates of associations of BMI with protein traits

In a linear regression model adjusted for age and sex among 2 729 adults, BMI (per SD higher) was associated with 1 576 proteins (39%) at the level P < 1.4 x 10^-5^ (multiple testing reference point) Supplementary Table 2). In a second model additionally adjusting for frequencies of smoking and alcohol intake among 2 380 adults, there were 1 447 associations at the same reference point (Supplementary Table 3). The strongest positive associations were with leptin (0.74 SD, 95% CI 0.71-0.77, P=9.9x10^-324^) and adipocyte fatty acid binding protein (FABP4) (0.58 SD, 95% CI 0.55-0.62, P=6.4x10^-211^). BMI (per SD) was also strongly positively associated with inflammatory proteins such as Complement Factor I (0.46 SD, 95% CI 0.43-0.50, P=5.6x10^-122^) and CRP (0.44 SD, 95% CI 0.41-0.48, P=8.2x10^-112^). BMI (per SD) also showed strong negative associations with proteins such as insulin-like growth factor-binding protein (IGFBP) 2 (-0.48 SD, 95% CI -0.51 to -0.44, P=2.7x10^-133^) and sex hormone-binding globulin (SHBG) (-0.43 SD, 95% CI -0.47 to -0.39, P=2.4x10^-106^).

### Observational associations of covariables with BMI and protein traits

Age, sex, and frequencies of smoking and alcohol intake were each associated with BMI (Supplementary Table 4). Males had a higher BMI than females (0.17 SD, 95% CI 0.10-0.25, P=5.8x10^-6^). Age was positively associated with BMI (0.01 SD higher per year older, 95% CI 0.009-0.015, P=1.2x10^-18^). Smoking frequency was positively associated with BMI, but alcohol intake frequency was negatively associated with BMI. Covariables (age, sex, smoking and alcohol) showed associations with protein traits (Supplementary Tables 5-8 and Supplementary Figures 4A-D). There was evidence for 18 associations between age and proteins, 26 associations between sex and proteins, 38 proteins associated with smoking and 137 proteins associated with alcohol at the Bonferroni-adjusted level of p<1.4x10^-5^.

### Associations of the GRS for BMI with measured BMI and covariables

The distribution of the GRS among participants was normal (mean=0.08, SD=0.29, W=0.99, P=0.73, N=2 729). The GRS was associated with BMI, explaining 2.8% of its variance (R^2^=0.028, P=1.6x10^-18^, Table 2). There was no strong evidence of association between GRS and age (R^2^=0.001 ‘ P=0.11) sex (R^2^=6x10^-5^, P=0.28) smoking frequency (R^2^=< 0.0001, P=0.91) or alcohol intake (R^2^<0.0001 P=0.44).

### MR estimates of associations between BMI and protein traits

**Figure 1:**
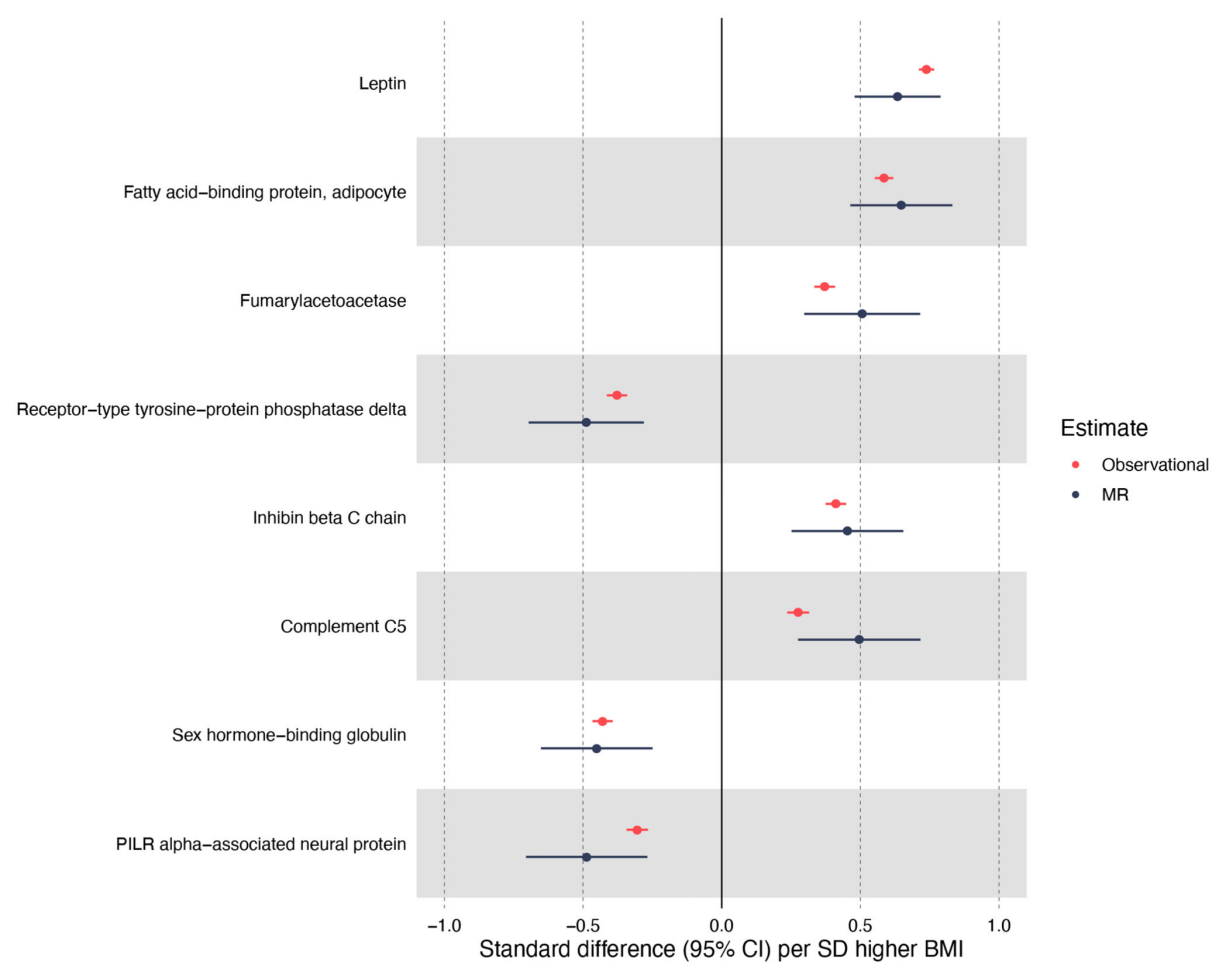
Strongest BMI and protein Mendelian randomisation associations with corresponding observational associations. Forest plot of MR results of BMI and protein traits based on P<1.4x10^-5^ and their corresponding observational estimates.

In MR analyses, eight unique BMI-protein associations were detected at the level P<1.4x10^-5^ (multiple testing reference point, Figure 1). MR estimates provide an estimate of the causal association between protein (in SDs) per SD higher BMI. The strongest association of BMI was again with leptin (0.63 SD, 95% CI = 0.48-0.79; P=1.6x10^-15^); this was followed by the association with FABP4 (0.65 SD, 95% CI = 0.46-0.83; P=6.7x10^-12^). A strong negative association was also seen between BMI (per SD) and SHBG (-0.45 SD, 95% CI -0.65 to -0.25, P=1.4x10^-5^). Other BMI-protein associations (P<1.4x10^-5^) included positive associations with fumarylacetoacetase, inhibin β C chain and complement C5, and negative associations with receptor-type tyrosine-protein phosphatase delta and PILR alpha-associated neural protein. Supplementary Table 9 provides the full MR results.

### Comparison of observational and MR estimates

The distribution of P-values for associations between BMI and protein traits suggested an overrepresentation of signal for the observational estimates of BMI and protein traits; far more than expected from chance alone (Supplementary Figure 5A). In contrast to this, the extent of this overrepresentation was reduced considerably in the MR (Supplementary Figure 5B).

**Figure 2:**
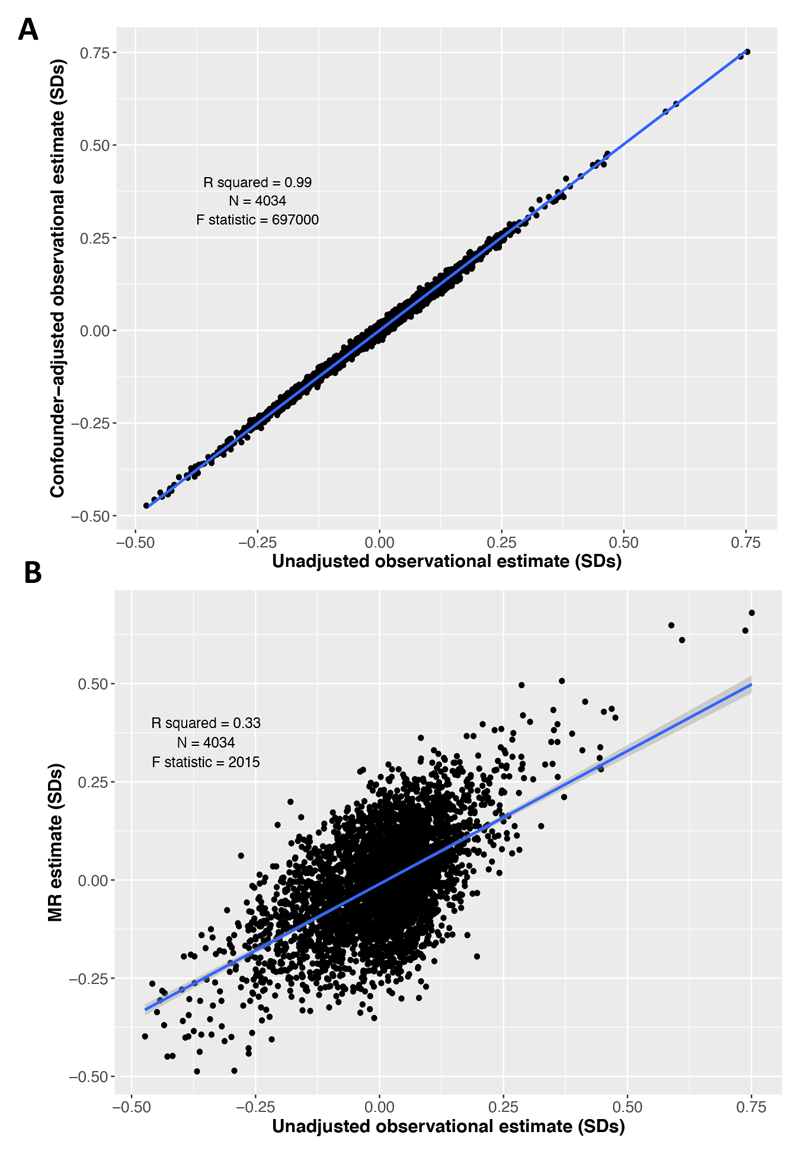
Observational and Mendelian Randomisation estimates show a positive association. **A**) Scatter plot of the unadjusted (age and sex adjusted) observational estimates and the confounder-adjusted observational estimates for BMI and protein traits with a regression line (blue). **B**) Scatter plot of the unadjusted (age and sex adjusted) observational estimates and the MR estimates for BMI and protein traits with a regression line (blue).

The unadjusted and confounder-adjusted regression coefficients for BMI and protein traits were strongly associated (β=0.99 SDs, R^2^=0.99, P=9.9x10^-324^ Figure 2A). Compared with the observational estimates, the MR estimates were less precise, but there was a strong positive association between the beta coefficients from observational and MR estimates (β=0.68 SDs, R^2^=0.33, P=9.9x10^-324^) (Figure 2B). After removing the proteins where P<1.4x10^-5^, the strength of association between unadjusted and adjusted observational estimates remained, but the association between observational and MR estimates attenuated slightly (Supplementary Figures 6A/B). These results suggest causal effects of BMI across the general proteome.

### Enrichment analysis of strongest BMI-protein associations

In examining the clustering of proteins, visual representation using a scree plot suggested there were five PCs that explained 30.3% of the variance (Supplementary Figure 3). After PC5 there was clear drop in variance explained, therefore other PCs were excluded. These five PCs were entered into a k-means analysis, which provided evidence for five clusters (grouping of individual proteins is included in Supplementary Table 9). To identify which cluster was most strongly affected by BMI, the median absolute beta coefficient divided by the SE for each cluster was compared with the overall estimate. Six of the proteins out of the eight strongest BMI-protein MR estimates were in cluster 2 (Supplementary Table 9). There was consistent evidence that cluster 2 showed a stronger association with BMI than the overall average BMI-protein effect both observationally (3.79 (IQR 1.62-7.06) vs 3.35 (IQR 1.57-5.83) respectively, P=3.7x10^-4^) and in MR (0.85 (IQR 0.41-1.46) vs 0.74 (IQR 0.32-1.14), P=5.3x10^-6^, Supplementary Table 10). Cluster 2 showed consistent evidence of a having the largest BMI effect. Compared with the full protein list in SomaLogic, the proteins in cluster 2 were enriched for disease (Table 3), including cardiovascular disease (1.14 fold enrichment, P=1.3x10^-4^), renal disease (1.22 fold enrichment, P=1.0x10^-3^) cancer (1.1 fold enrichment, P=9.5x10^-3^) and metabolic disease (1.08 fold enrichment, P=4.2x10^-2^). No other individual cluster showed enrichment for disease. Enrichment for disease was also explored by comparing the proteins which had an association with BMI (P<1.4x10^-5^) in the confounder-adjusted regression model with the total protein list. Compared with the full protein list, the proteins which showed a stronger observational association with BMI were enriched for renal disease (1.21 fold enrichment, P=0.001) and metabolic disease (1.9 fold enrichment, P=0.015, Supplementary Table 11).

## Discussion

This study sought to estimate the effects of adiposity on a comprehensive set of protein traits only recently measurable by untargeted proteomics using observational and MR methods. Observational results provided evidence for associations between BMI and 1 576 proteins and MR was performed to reduce confounding. MR results suggest that BMI alters protein traits involved in regulating appetite, sex hormones, inflammation, and other systems; specific proteins most altered by BMI include leptin, FABP4, and SHBG. Results of follow-up analyses suggest that the cluster of proteins most altered by BMI is enriched for genes associated with cardiovascular and metabolic disease.

This study explored the effect of BMI on a large set of circulating proteins in an MR framework. Previous studies have used observational epidemiology to explore the effect of obesity on the plasma proteome: one study used mass spectrometry and found an increase in Complement Factors I, B and H and an increase in CRP (18). These findings were replicated in our current observational analysis using the SomaLogic platform, indicating that associations are detectable across different proteomic platforms. The only association that did not replicate in the current study was the positive association with protein S100-A9. Although the MR analysis did not support some of these BMI-protein associations as being causal based on a P-value reference point, the strong association between the observational and MR estimates throughout the entire effect distribution suggests that disagreements between methods are likely an issue of power given current sample sizes.

Previous work implementing MR to examine the relationship between BMI and ~1000 proteins (measured using the same SomaLogic array) provided corroborative evidence to that shown here (34). Both studies suggested a positive association between BMI and leptin, as well as a negative association with SHBG. Other proteins, such as IGFBP1/2 and growth hormone receptor, did not pass our multiple testing threshold, but the direction and magnitude of estimates were in agreement, suggesting a possible causal effect that was not detectable in the current study. Building on previous work, the current study provides MR estimates for >3600 proteins, offering a wider proteomic profile and detecting additional associations such as that between BMI and fumarylacetoacetase and inhibin β C chain. Furthermore, the inclusion of over threefold more proteins allowed a more comprehensive enrichment analysis to be performed.

For proteins with stronger MR derived association evidence, it is important to explore whether they have a potential role in disease. Identification of individual proteins could help to guide future intervention if changes in proteins can be mapped to disease outcomes. Our results suggest a strong positive effect of BMI on levels of leptin, a hormone released by white adipose tissue which suppresses appetite (35). The direction of effect agrees with estimates from previous cross-sectional and MR studies (16, 36), indicating leptin receptor resistance (37). There is observational evidence in humans that higher leptin can induce greater aggregation of platelets (cells involved in haemostasis) (38). In a larger observational study, leptin was found to be associated with higher risk of coronary events independent of BMI (39).

Our results help to provide contextualisation for proteins which have already been implicated in disease. For example, results suggest a strong positive effect of BMI on FABP4, an adipokine found primarily in adipocytes and macrophages (40). This MR estimate supports the association which has been suggested in previous observational studies (41). FABP4 has been implicated in cardiometabolic disease: a SNP which increases FABP4 was found to raise the odds of type II diabetes among adults (42), potentially through its contribution to higher insulin resistance (43). FABP4 has also been associated with higher risk of atherosclerosis among adults (44). A strong SHBG-lowering effect of higher BMI was also suggested here. The SHBG molecule is a glycoprotein which binds androgens and oestrogens and suppresses their activity (45); a reduction in SHBG is therefore expected to lead to higher levels of circulating sex hormones. The negative effect of BMI on SHBG seen here supports observational findings (46) (47) (48). When evaluating the role of SHBG in disease, MR analysis suggests that an increase in SHBG contributes to a decrease in risk of cardioembolic stroke (49). Other studies have also implicated lower SHBG levels in increasing type II diabetes risk (42, 50). The exact mechanisms leading from decreased SBHG to ill-health is unclear, but may arise as a result of the increased bioavailability of testosterone and oestrogen (45).

Despite these possible protein involvements in cardiometabolic disease, it remains difficult to assess the contribution of individual proteins as they are not entirely independent and any pathological effects would likely be due to a global change in protein composition. There are not distinct groupings in the SomaLogic data as there often are with, for example, metabolomics data. We therefore examined proteins grouped into clusters of similar features, compared BMI-protein estimates of each cluster with overall estimates and explored enrichment for genes related to disease. The cluster most altered by BMI (cluster 2) included most of the eight proteins with the strongest BMI effects from MR analyses, as well as various complement factors, chemokines and coagulation factors, and was found to be enriched for genes related to cardiovascular disease, renal and metabolic diseases, and cancer. Enrichment was similar when comparing the proteins that had an observational association with BMI with all proteins included, with enrichment appearing greatest for renal and metabolic disease. Together, this suggests that changes in proteins may mediate effects of obesity on cardiometabolic diseases; more focused investigations of these proteins are now needed.

This study has some limitations. Firstly, although INTERVAL is one of the largest existing cohorts to have untargeted proteomic data based on the SomaLogic platform, the sample size is still relatively modest and may have low power to detect some associations when using MR: based on the detectable (P<1.4x10^-5^) median absolute observational effect size (0.13 SDs), our analyses had 80% power to detect MR effect sizes >= 0.33 SDs (α=0.05) for our sample size (N=2737) (51). With greater statistical power, there would likely be more proteins detected with MR. This was reinforced by the high agreement in the magnitude of effect estimates seen in observational and MR analyses which applied throughout the effect distribution. Secondly, height and weight were self-reported which could bias results towards the null due to systematic error in BMI measurement. However, strong correlations are often reported between self-reported and measured BMI (52) and the validity of self-reported BMI is supported by the association between the GRS for BMI and self-reported BMI in INTERVAL to the degree expected. Thirdly, the small degree of overlap between INTERVAL and UK Biobank (participants used for the source GWAS for BMI who were also in INTERVAL) may have a biasing effect on estimates, though this is likely to be towards the null. We anticipate that overall, this bias would make estimates more conservative (53). Fourth, we recognise a lack of availability of possible confounders such as socio-economic position (which likely affect both BMI and protein traits related to cardiovascular disease processes (54, 55)). Residual confounding may help account for the divergence between observed and expected P-values seen in observational versus MR models. Fifth, the proteins examined are highly correlated and we therefore may not fully be describing changes in individual proteins. Evidence from case-control cohorts as well as functional and animal studies would help isolate individual proteins that are altered and contribute to disease. Finally, although analyses provide insight into the proteomic effects of BMI, it does not distinguish between the type of adiposity. It would be useful to distinguish between the effects of subcutaneous and visceral fat using dual-energy X-ray absorptiometry (DXA) derived measurements, but these were not available in the INTERVAL dataset.

This study utilised SomaLogic to explore the relationship between BMI and plasma proteins in unprecendented scope and detail, in both an observational and MR framework. We provide evidence for a broad impact of higher adiposity on the human proteome. Causal evidence was strongest for BMI in relation to proteins involved in regulating appetite, sex hormones, and inflammation. Identification of BMI-driven protein changes could provide therapeutic targets for prevention of obesity-related disease. Protein alterations were also found to be enriched for genes related to cardiovascular and metabolic disease. Altogether, these results help to focus attention onto new potential proteomic signatures of obesity-related disease. Further characterisation of the role of such proteomic profiles in disease using MR is warranted.

## Supplementary Material

Supplementary Figures

Supplementary Tables

## Figures and Tables

**Table 1:** Characteristics of included participants

Variable		Mean (SD) or %	N
**Age (years)**		45.0 (14.1)	2,737
**Sex**	**Male**	51.7 %	2,737
	**Female**	48.3 %	
**Weight (kg)**		78.5 (16.1)	2,736
**Height (cm)**		172.7 (9.7)	2,730
**Body mass index (kg/m^2^)**		25.9 (4.8)	2,729
**Smoking frequency**	**Never**	59.1 %	2,675
	**Occasional**	11.9 %	
	**Most days or every day**	29.0 %	
**Alcohol intake frequency**	**Rarely**	11.7 %	2,422
	**Less than once a week**	16.9 %	
	**One or two times a week**	37.4 %	
	**Three to five times a week / most days**	34.1 %	

**Table 2:** Associations of the genetic risk score for BMI with reported BMI and covariables

Variable	N	Beta coefficient (per 1-unit increase in GRS)	Standard error	P value	Adjusted R^2^	F statistic
**BMI (SDs)**	2729	0.57	0.06	1.64x10^-18^	0.028	78.20
**BMI (kg/m^2^)**	2729	2.54	0.31	4.82x10^-16^	0.024	66.68
**Age (years)**	2737	-1.47	0.92	0.11	0.001	2.55
**Sex (1 = female, 2 = male)**	2737	-0.04	0.03	0.28	5.96E-05	1.16
**Smoking frequency (1 = never, 2 = occasional, 3 = most days or every day)**	2675	-0.02	0.14	0.91	-0.0004	0.01
**Alcohol intake frequency (1 = rarely, 2 = < 1 a week, 3 = 1-2 times a week, 4 = most days or every day)**	2422	-0.06	0.07	0.44	-0.0002	0.60

**Table 3:** Cluster 2 vs full SomaLogic protein enrichment results for disease class using DAVID bioinformatics 6.8 (31)

Term (Genetic Association Database disease class)	Count	%	List Total	Population Hits	Population Total	Fold Enrichment	P Value	Bonferroni-adjusted P value
**Cardiovascular**	439	34.1	1024	1023	2723	1.14	7.3E-06	1.3E-04
**Renal**	216	16.8	1024	472	2723	1.22	5.8E-05	1.0E-03
**Cancer**	401	31.2	1024	958	2723	1.11	5.3E-04	9.5E-03
**Pharmacogenomic**	357	27.8	1024	856	2723	1.11	1.9E-03	3.4E-02
**Metabolic**	504	39.2	1024	1243	2723	1.08	2.4E-03	4.2E-02
**Vision**	100	7.8	1024	216	2723	1.23	5.9E-03	1.0E-01
**Haematological**	168	13.1	1024	388	2723	1.15	9.8E-03	1.6E-01
**Reproduction**	152	11.8	1024	351	2723	1.15	1.4E-02	2.3E-01
**Immune**	360	28.0	1024	886	2723	1.08	1.5E-02	2.4E-01
**Neurological**	311	24.2	1024	759	2723	1.09	1.6E-02	2.5E-01
**Aging**	117	9.1	1024	278	2723	1.12	7.5E-02	7.5E-01
