# Supplementary material for: Effects of adiposity on the human plasma proteome: Observational and Mendelian randomization estimates
Source: Int J Obes (Lond). Author manuscript; Available in PMC 2021 Sep 24. (PMC8455324; doi:10.1038/s41366-021-00896-1)
Supplement: Supplementary Figures [file EMS128858-supplement-Supplementary_Figures.docx]

**Supplementary Figures**

**
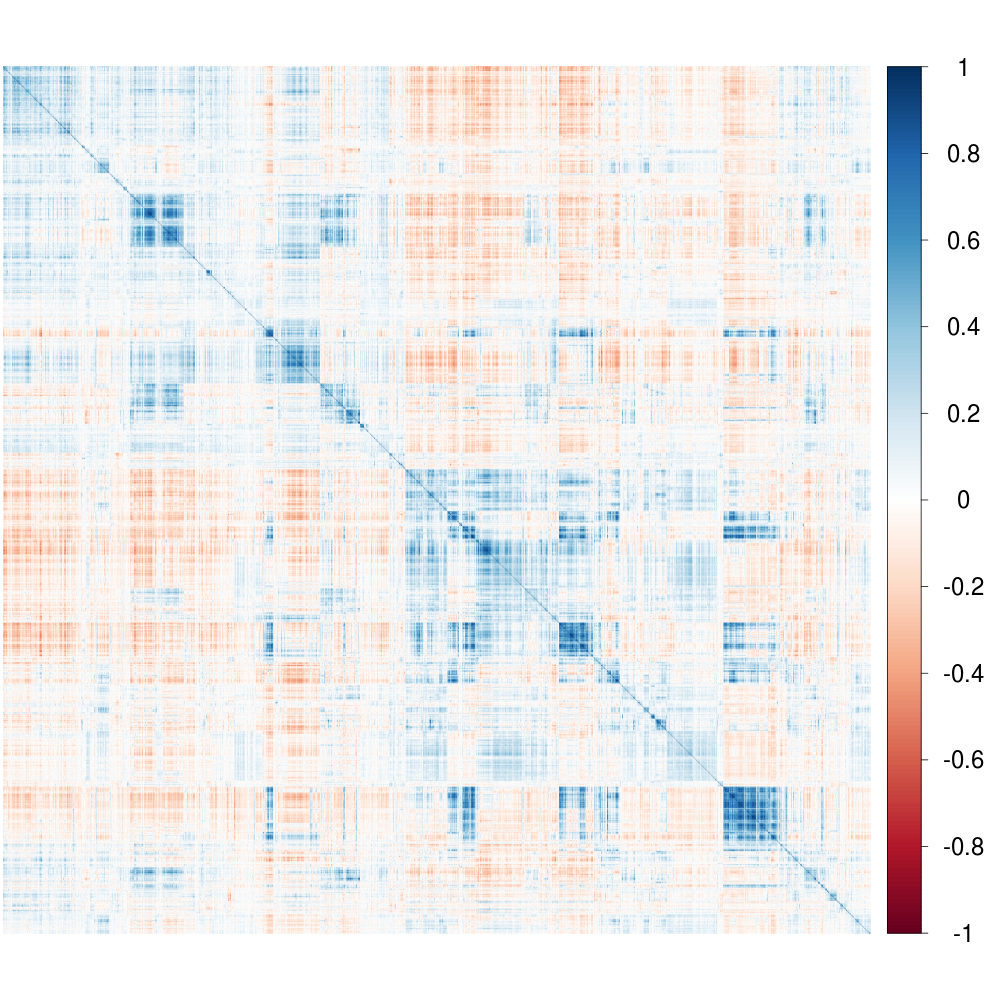
**

**Supplementary Fig 1**. Correlation matrix of all protein traits (4,034) where the colour corresponds to the correlation coefficient ranging from 1 (dark blue) to -1 (dark red).

**Supplementary Fig 2.** Cluster dendrogram showing the hierarchical relationship between protein levels. Height is calculated as (1-correlation coefficient). At a height of 0.2 and 0.4 (red dashed lines), the number of independent proteins was 3,655 and 3,016 respectively.

F

E

D

C

B

A

**Supplementary Fig 3**. **Principal component analysis (PCA) and k-means clustering provides evidence for five clusters**

A-D) Principal component (PC) 1 vs PC2-PC5 for the study protein data. Each dot represents a protein, and the colors represent the clusters identified by the k-means analysis (1=black, 2=red, 3=green, 4=dark blue, 5=light blue, 6=pink). E) PC scree plot displaying the proportion of variance explained by each of the first 20 PCs. F) K-means scree like plot displaying the variance explained by clusters for each of the 19 k-means analysis, where in each analysis k (the number of clusters) was set from two to 20.

B

A

C
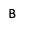


D

**Supplementary Fig 4.** Q-Q plots for the expected against observed -log_10_(p) values for the association of age (**A**), sex (**B**), smoking (**C**) and alcohol (**D**) with protein traits

A

B

**Supplementary Fig 5. A**) Q-Q plot of expected against observed -log_10_(p) values for the unadjusted observational BMI-protein trait estimates **B**) Q-Q plot of expected against observed -log_10_(p) values for the Mendelian Randomization BMI-protein trait estimates.

A

B

**Supplementary Fig 6. A)** Scatter plot of the unadjusted (age and sex adjusted) observational estimates and the confounder-adjusted observational estimates for BMI and protein traits with a regression line (blue), with the top eight MR BMI-associated proteins excluded. **6B**) Scatter plot of the unadjusted (age and sex adjusted) observational estimates and the MR estimates for BMI and protein traits with a regression line (blue), with the top eight MR BMI-associated proteins excluded.
